# Supplementary material for: Seminoma and Embryonal Carcinoma Footprints Identified by Analysis of Integrated Genome-Wide Epigenetic and Expression Profiles of Germ Cell Cancer Cell Lines
Source: PLoS One. 2014 Jun 2;9(6):e98330. doi: 10.1371/journal.pone.0098330 (PMC4041891; doi:10.1371/journal.pone.0098330)
Supplement: File S1 — ZIP file containing DMRforPairs output for significant regions. Please start from the html files. (ZIP) [file pone.0098330.s008.zip › figures/5432.pdf]

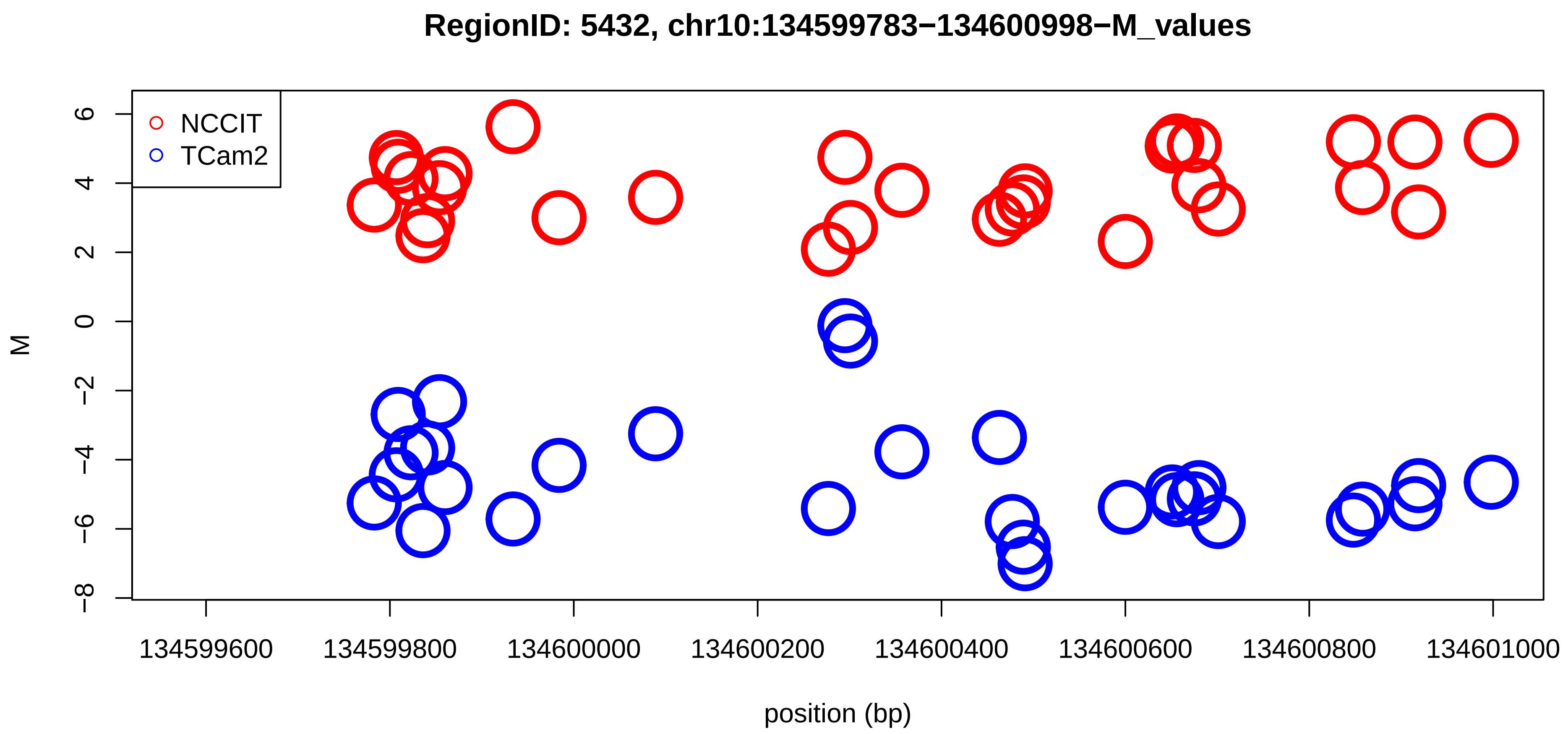
RegionID: 5432, chr10:134599783−134600998−M_values
NCCIT
TCam2
M
6
4
2
0
−2
−4
−6
−8
134599600
134599800
134600000
134600200
134600400
134600600
134600800
134601000
position (bp)

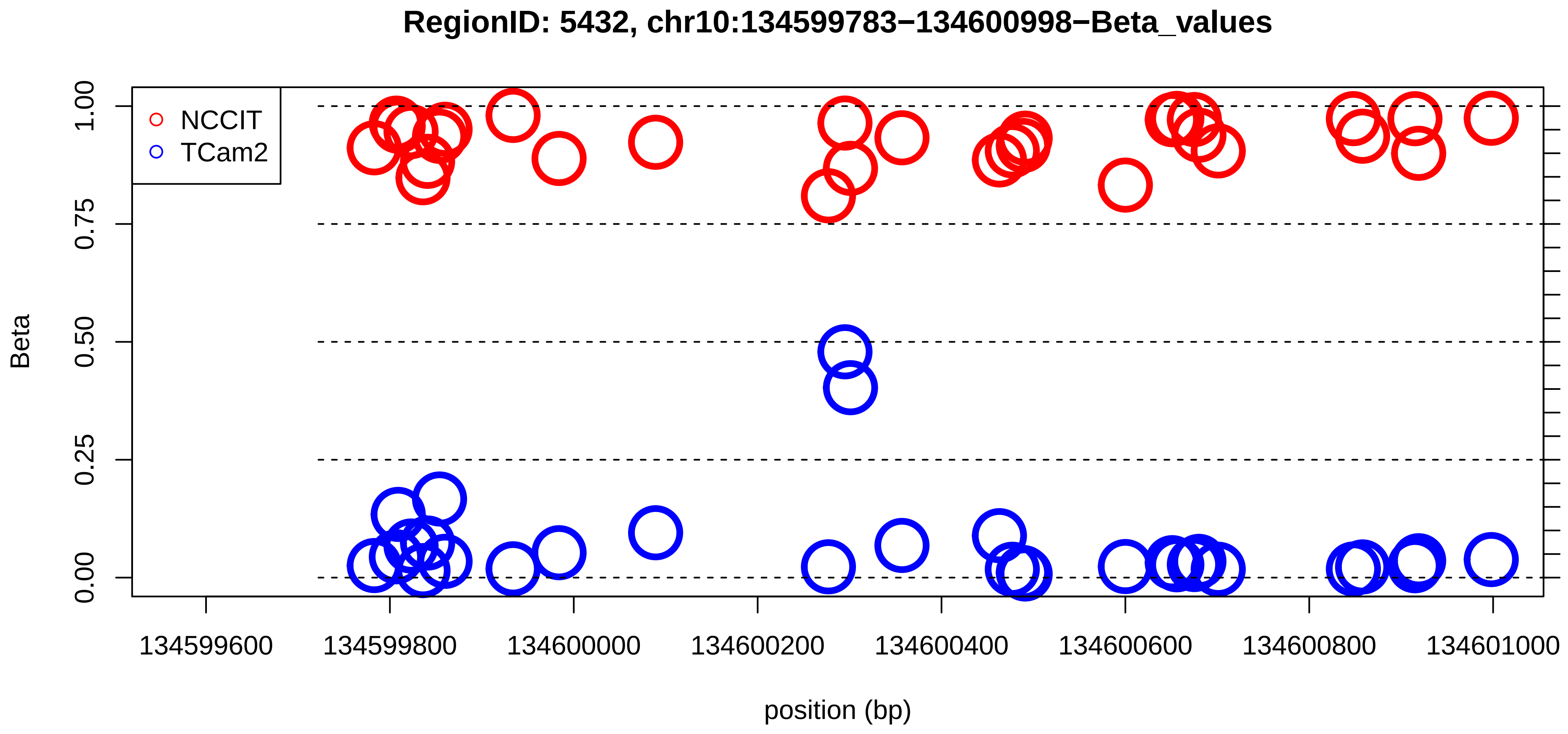
RegionID: 5432, chr10:134599783−134600998−Beta_values
NCCIT
TCam2
Beta
1.00
0.75
0.50
0.25
0.00
134599600
134599800
134600000
134600200
134600400
134600600
134600800
134601000
position (bp)

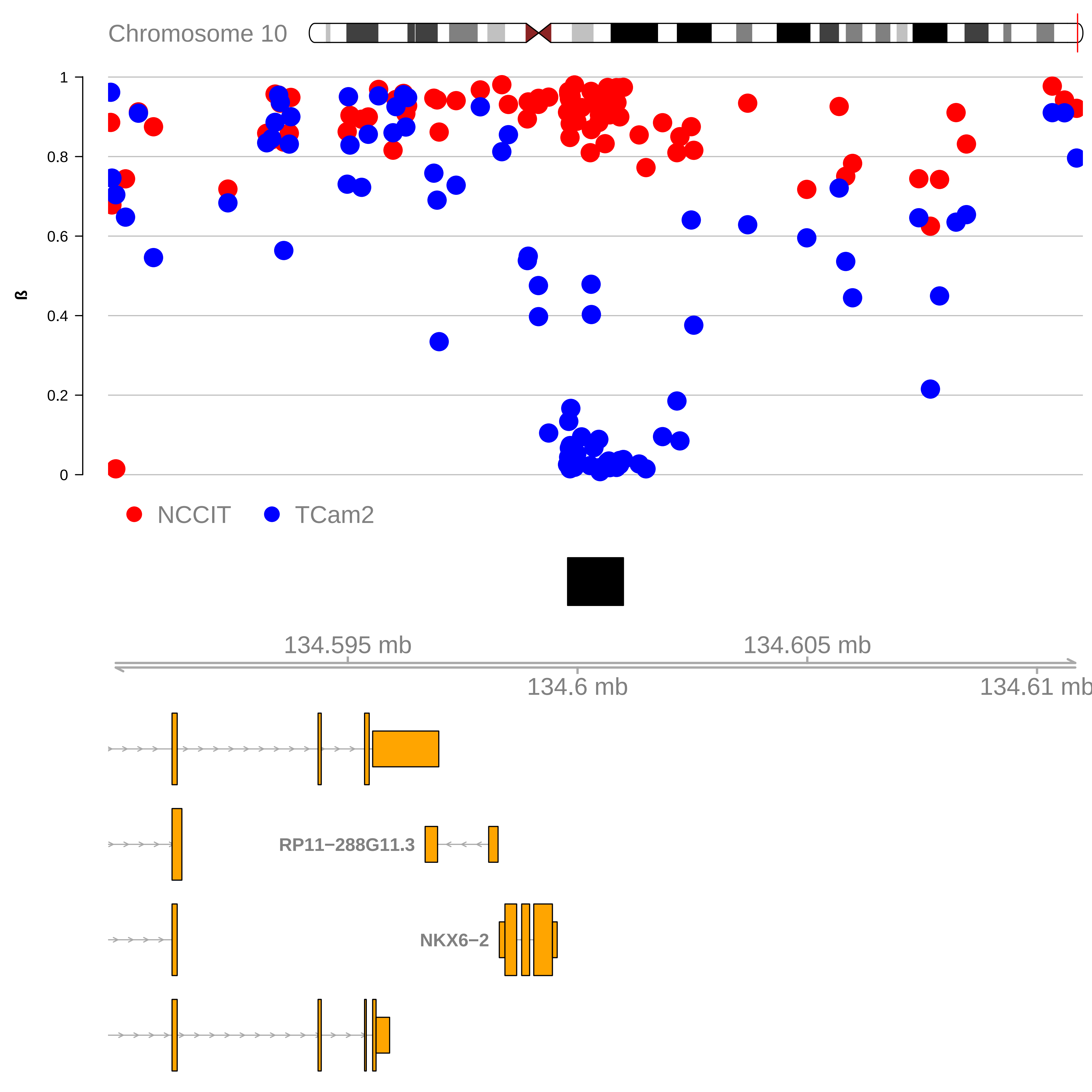

Chromosome 10
1
0.8
0.6
0.4
0.2
0
ß
NCCIT
TCam2
134.595 mb
134.605 mb
134.6 mb
134.61 mb
RP11−288G11.3
NKX6−2
